# 448. Highs and Lows: Changes in National and Regional Patterns of Injection Drug Use Associated Endocarditis from 2016-2022

**DOI:** 10.1093/ofid/ofaf695.147

**Published:** 2026-01-11

**Authors:** Scott A Fabricant, Paul Christine, Simeon D Kimmel

**Affiliations:** Boston Medical Center, Boston, Massachusetts; University of Colorado Anschutz, Aurora, Colorado; Boston University School of Medicine and Boston Medical Center, Boston, Massachusetts

## Abstract

**Background:**

Infective endocarditis (IE) is among the most dangerous complications of injection drug use. IE related to injection drug use (IDU-IE) in the United States increased dramatically alongside the opioid overdose crisis. Drug use patterns have since evolved, with widespread fentanyl, rising methamphetamine and other stimulant use, falling overdose rates, and shifts towards more smoking or sniffing of drugs. The effect of these changes on IDU-IE hospitalizations is currently not known.

**Figure:**
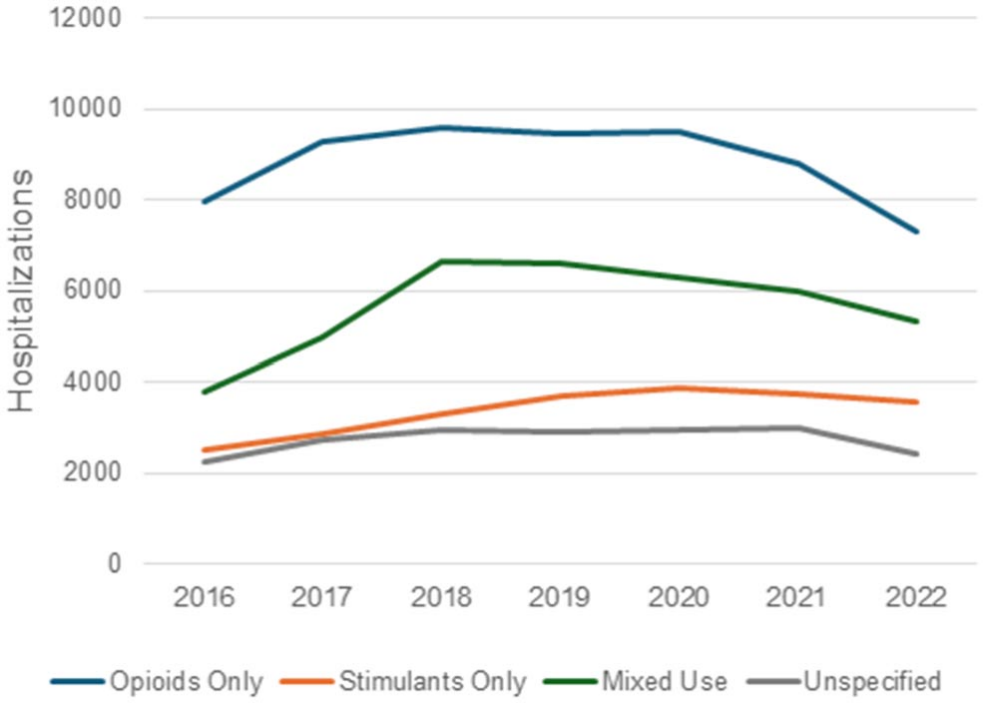
Hospitalizations for IDU-IE by Drug Type Estimated annual hospitalizations for injection drug use associated infective endocarditis (IDU-IE) in the United States, stratified by type of drug use coded in admission – opioids only, stimulants only, both opioids and stimulants (mixed use), or drug-related codes that do not specify substance (unspecified)

**Figure:**
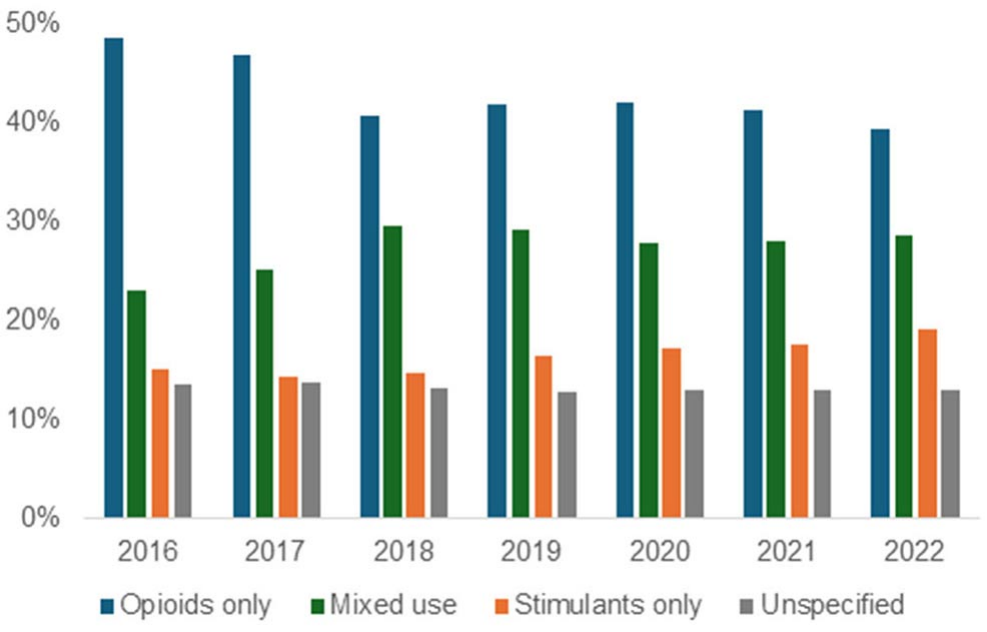
Proportion of IDU-IE by Drug Type Proportion of estimated annual hospitalizations for injection drug use associated infective endocarditis (IDU-IE) in the United States, stratified by type of drug use coded in admission – opioids only, stimulants only, both opioids and stimulants (mixed use), or drug-related codes that do not specify substance (unspecified)

**Methods:**

We examined IDU-IE trends from 2016 through 2022 using the National Inpatient Sample, a representative sample of United States hospitalizations. We identified hospitalizations of individuals aged 18 and above with ICD-10 codes for IE, and used a previously published list of ICD-10 codes related to drug use to identify patients whose IDU-IE was likely caused by injection drug use. We examined number of IDU-IE hospitalizations and proportion of IE attributable to specific types of drug use, taking into account complex survey design, to generate national and regional weighted estimates for each year.

**Figure:**
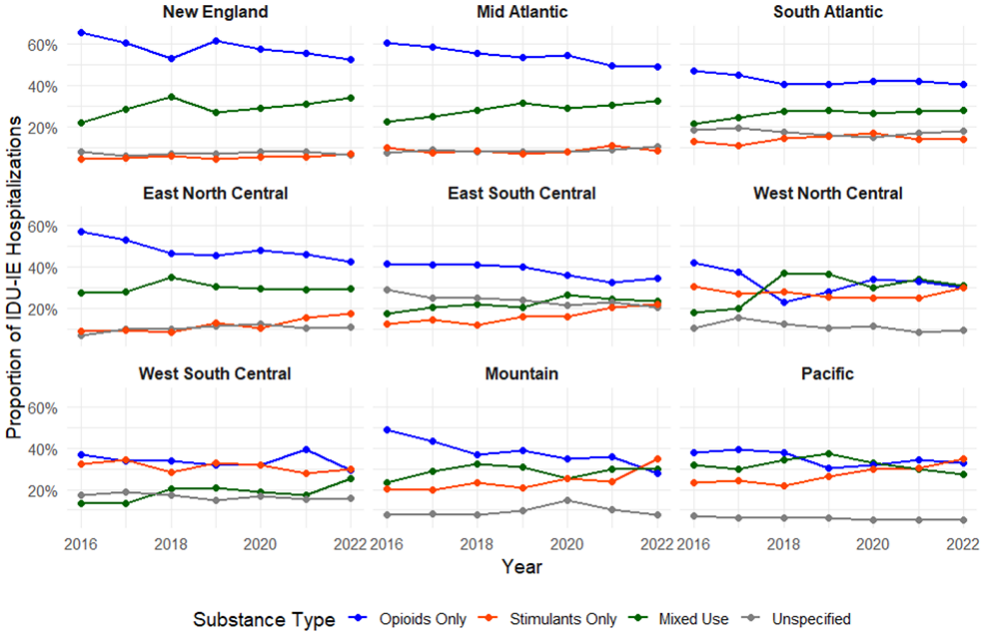
Proportion of IDU-IE by Drug Type across US Regions Proportion of estimated annual hospitalizations for injection drug use associated infective endocarditis (IDU-IE) in the 9 major geographic areas of the United States, stratified by type of drug use coded in admission – opioids only, stimulants only, both opioids and stimulants (mixed use), or drug-related codes that do not specify substance (unspecified)

**Results:**

There were estimated 144,090 IDU-IE hospitalizations between 2016 and 2022. They peaked in 2019 with 22,695 hospitalizations, with an increasing rate of decline each year after (down 13.1% between 2021 and 2022). IDU-IE associated with types of drug use codes has changed over this period: opioid alone decreased from 48.5% to 39.2% of all IDU-IE, stimulant alone increased from 15.1% to 19.2%, and mixed use of stimulants and opioids increased from 22.9% to 28.6%; substance-nonspecific drug use codes remained stable at 13.2±0.6%. Regionally, IDU-IE hospitalizations involving stimulants overtook opioids as the leading involved substance in Pacific, Mountain, and West Central regions.

**Conclusion:**

IDU-IE peaked in 2019 and declined through 2022, with greater reduction in hospitalizations involving opioid use compared to stimulant use. Consequently, the proportion of IDU-IE related to stimulant use has been rising, most sharply in people who use stimulants alone. This trend was most pronounced in the West, where IDU-IE involving stimulants surpassed those involving opioids. Understanding these shifts and their causes can help allocate prevention and treatment efforts.

**Disclosures:**

All Authors: No reported disclosures

